# Optical and Photocatalytic Properties of Cobalt-Doped LuFeO_3_ Powders Prepared by Oxalic Acid Assistance

**DOI:** 10.3390/molecules28155730

**Published:** 2023-07-28

**Authors:** Zhi Wang, Changmin Shi, Pengfei Li, Wenzhu Wang, Wenzhen Xiao, Ting Sun, Jing Zhang

**Affiliations:** 1 School of Physics and Electrical Engineering, Anyang Normal University, Anyang 455000, China; 2 School of Physics and Electronic Engineering, Linyi University, Linyi 276000, China

**Keywords:** cobalt doping, rare-earth orthoferrites, optical properties, photocatalysis, hole scavengers

## Abstract

B-site cobalt (Co)-doped rare-earth orthoferrites ReFeO_3_ have shown considerable enhancement in physical properties compared to their parent counterparts, and Co-doped LuFeO_3_ has rarely been reported. In this work, LuFe_1−x_Co_x_O_3_ (x = 0, 0.05, 0.1, 0.15) powders have been successfully prepared by a mechanochemical activation-assisted solid-state reaction (MAS) method at 1100 °C for 2 h. X-ray diffraction (XRD) and Fourier transform infrared (FTIR) spectroscopy studies demonstrated that a shrinkage in lattice parameters emerges when B-site Fe ions are substituted by Co ions. The morphology and elemental distribution were investigated by scanning electron microscopy (SEM) and energy dispersive spectroscopy (EDS). The UV–visible absorbance spectra show that LuFe_0.85_Co_0.15_O_3_ powders have a narrower bandgap (1.75 eV) and higher absorbance than those of LuFeO_3_ (2.06 eV), obviously improving the light utilization efficiency. Additionally, LuFe_0.85_Co_0.15_O_3_ powders represent a higher photocatalytic capacity than LuFeO_3_ powders and can almost completely degrade MO in 5.5 h with the assistance of oxalic acid under visible irradiation. We believe that the present study will promote the application of orthorhombic LuFeO_3_ in photocatalysis.

## 1. Introduction

In recent decades, a flurry of research has been devoted to studying perovskite-type rare-earth ferrites, RFeO_3_ (R denotes rare-earth), due to their potential applications in data storage devices [1,2], catalysis [3,4], solid-oxide cells [5], gas sensors [6], etc. Since the ABO_3_ perovskite structure has a large tolerance for structural distortion [7], the electrical, optical, and magnetic properties can be effectively modified via tuning spin, charge, orbital, and lattice coupling by substituting A- and/or B-sites to meet the desired demands in applications, which makes RFeO_3_ compounds rather appealing [8].

Among these applications, photocatalysis is generally referred to as the catalysis of a photochemical reaction at a solid surface, usually a semiconductor. For light-harvesting and visible light-driven photocatalysis, the photocatalytic capacity can be improved by bandgap engineering via doping [9], providing more active sites via the design of active nanostructures [5], preventing electron–hole recombination via involving electron–hole scavengers [3], enhancing adsorption via introducing vacancies [10,11], etc. Bandgap engineering via doping is challenging in these approaches. Firstly, the preparation method should be effective in replacing the atoms at the target sites. Secondly, semiconductor doping can simultaneously alter its bandgap and electronegativity, which determine the redox potential at the conduction band minimum (CBM) and the valence band maximum (VBM) [12], and the redox potential plays a key role in the photocatalysis process.

LuFeO_3_, as an important member of the ReFeO_3_ family, is valued for its stability, high Neel temperature, large magnetocrystalline anisotropy, and Dzyaloshinskii–Moriya interaction [13,14,15]. More recently, Zhou et al. [3] reported orthorhombic LuFeO_3_ as a new photocatalyst for dye degradation. They pointed out that the reaction of holes with OH^−^ is possibly not the dominant way to generate the free radical ·OH. This increases the possibility of improving catalytic efficiency via doping because the effect of VBM oxidation potential can be weakened to some extent.

Metal doping is a well-known strategy to enhance the electrical, chemical, and optical performance of materials, such as the durability of Li-ion batteries [16], the pseudocapacitive activities of WS_2_ [17], the nonlinear optical response of aluminum nitride nanocages [18], the hydrogen evolution reduction of biomass-based carbon materials [19], the electrochemical activities of MnO_2_ [20], etc. Among these metals, cobalt (Co) is considered an interesting dopant because of its high Pauling electronegativity number (1.88) [21], various oxidation levels, and spin states [22,23]. Recently, researchers have carried out extensive investigations on the structure, morphology, and electrical, optical, and magnetic properties of B-site cobalt-doped orthorhombic RFeO_3_. Most research focuses on orthorhombic NdFeO_3_ [24,25,26,27,28], YbFeO_3_ [29], DyFeO_3_ [30], and LaFeO_3_ [31]. It has been found that these Co-doped RFeO_3_ systems show considerable enhancement in their physical properties compared to their parent counterparts. However, as orthorhombic LuFeO_3_ has the smallest tolerance factor (0.866) [32], its perovskite structure is the most unstable in RFeO_3_, so it is difficult to prepare B-site Co-doped LuFeO_3_, which has rarely been reported. In addition, there are few reports on the elimination of impurity phases in the preparation of Co-doped RFeO_3_, although it is important to eliminate the influence of impurities on the properties of pure-phase ReFeO_3_ systems.

The powders prepared by the solid-state reaction method, which usually results in large particle sizes and poor morphology, are seldomly employed to perform photocatalytic experiments. However, the mechanochemical activation-assisted solid-state reaction (MAS) method, a modified solid-state reaction method reported in our previous work [33], enables the preparation of desired powders at a relatively low temperature for a relatively short time. In this paper, we prepared B-site Co-doped LuFeO_3_ powders by the MAS method reported in our previous work [33]. The preparation, fine structure, morphology, and optical properties are investigated by X-ray diffraction (XRD), Fourier transform infrared spectroscopy (FTIR), scanning electron microscopy (SEM), energy dispersive spectroscopy (EDS), and UV–visible absorbance spectroscopy. The photocatalytic capacity was investigated by degrading methyl orange (MO) assisted by oxalic acid (OA) under visible light irradiation. A possible degradation mechanism is proposed.

## 2. Results and Discussion

### 2.1. Preparation of Co-Doped LuFeO_3_ Powders and Structure Analysis

**Figure 1:**
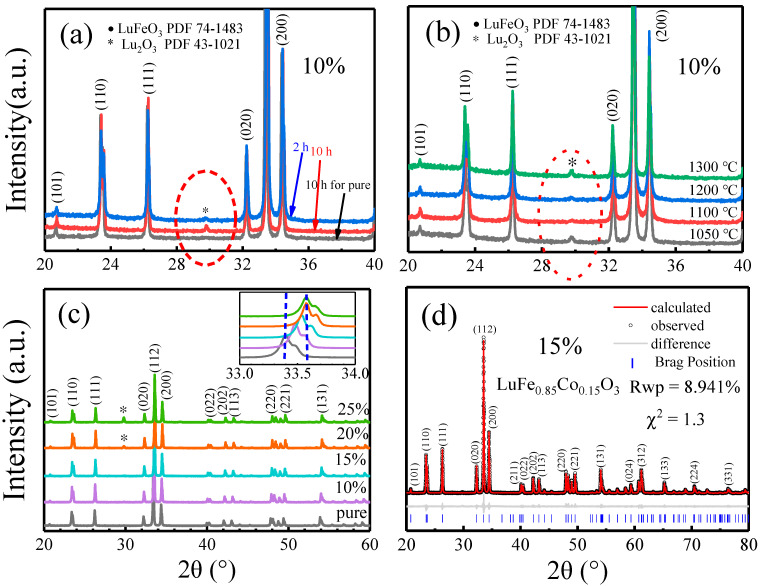
XRD pattern evolution of (**a**) 10% Co doping and calcination time at 1200 °C, (**b**) calcination temperature, and (**c**) Co doping amount (the upper right corner shows the high magnification XRD peak at the (112) plane). (**d**) Refinement of LuFe_0.85_Co_0.15_O_3_ by TOPAS.

The effects of Co doping amount, calcination time, and temperature on lutetium ferrite phase evolution were studied by XRD, and the optimum preparation parameters were obtained. The results are represented in Figure 1. As shown in Figure 1a, the LuFe_0.9_Co_0.1_O_3_ sample (marked in red) was attempted to be prepared using the same calcination as our previous work, at 1200 °C for 10 h [33], but a distinct peak at 29.8° corresponding to the impurity Lu_2_O_3_ was observed, as marked with a red ellipse. When the calcination time was reduced to 2 h (marked in blue), the content of Lu_2_O_3_ decreased significantly, which indicates that Co doping enables the system to be prepared at a lower energy level. Thus, the effects of the calcination temperature on the phase evolution of the lutetium ferrite system were studied by increasing the reaction temperature from 1050 °C to 1300 °C, as shown in Figure 1b. It can be seen that a low or high calcination temperature is not beneficial to obtaining the target product. The optimum preparation parameters for the LuFe_0.9_Co_0.1_O_3_ sample are calcination at 1100 °C for 2 h. Under this condition, a series of LuFe_1−x_Co_x_O_3_ (x = 0, 0.1, 0.15, 0.2, 0.25) samples were attempted to be synthesized, and the results are shown in Figure 1c. It can be observed that the peak at 29.8°, corresponding to Lu_2_O_3_, appears when x reaches 0.2. As mentioned in the Introduction, because the perovskite structure of orthorhombic LuFeO_3_ is most unstable in ReFeO_3_ systems, the Co doping level should be lower than in other ReFeO_3_ systems such as NdFeO_3_ [28] and DyFeO_3_ [30]. Further, the XRD patterns in Figure 1c indicate that the 2θ angle values of the (020), (112), and (200) diffraction peaks gradually shift to a higher angle after Co was included in the LuFeO_3_ lattice when no Lu_2_O_3_ peak emerged in the samples. The highlighted (112) plane shows an obvious change in the peak position. The d-spacings of the Co-doped LuFeO_3_ lattice should be narrowed by Co doping, according to Bragg’s law. Thus, a shrinkage in lattice parameters is expected.

To quantitatively describe the changes in lattice parameters by Co doping, the XRD pattern of a LuFe_0.85_Co_0.15_O_3_ (LC15) sample with a pure phase was processed by the Rietveld method for refinement using the Topas program, which is a reliable technique that can provide structural details of the materials [34]. The structure was successfully fitted by the perovskite structure with space group Pbnm, as depicted in Figure 1d. All diffraction peaks are well indexed, and no trace of any impurity peak is observed. The weighted profile residual Rwp and goodness of fit χ^2^ are 8.941% and 1.3, respectively, indicating a good agreement between the observed and calculated diffraction patterns [35]. All the Rietveld parameters, as obtained from the refinement, plus the counterparts of the parent LuFeO_3_ (LFO) [33], are tabulated in Table 1. A shrinkage in lattice parameters and volume of LC15 compared with that of LFO is observed. Since the ionic radius of Co^3+^ (54.5 pm in LS and 61.0 pm in HS) is slightly less than that of Fe^3+^ (64.5 pm in HS), shrinkage is expected, indicating a successful substitution of Co ions at Fe sites [17,36,37]. Similar results have also been reported for NdFe_1−x_Co_x_O_3_ systems [24,25,26].

**Figure 2:**
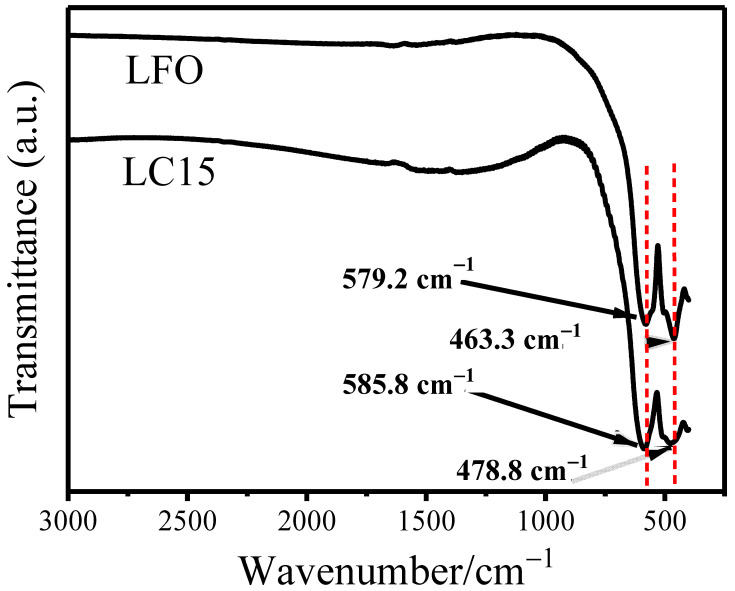
Infrared spectra of the samples LFO and LC15.

In order to explore the effect of Co doping on the fine structure of the parent LFO, we performed FTIR spectroscopy, an analytical technique capable of revealing the nature of structures by detecting lattice vibrations. The FTIR spectra were recorded in transmission geometry using a KBr disc in the range of 450–3000 cm^−1^, as illustrated in Figure 2. As per Rao [38], two major bands can be observed at 250–600 cm^−1^, which are the characteristic bands for rare-earth ferrites. These two bands are attributed to the Fe-O stretching and bending vibrations of the FeO_6_ octahedral groups in the perovskite compounds [38,39]. In our case, the two bands of LFO are located at 579.2 and 463.3 cm^−1^, while the counterparts of LC15 blueshift to 585.8 and 478.8 cm^−1^, respectively. As is well known, the wavenumber of IR bands can be influenced by atomic mass and bond length. However, there is little difference in atomic mass between cobalt (58.9) and iron (55.8) here, so it is reasonable to consider that the bond length of Fe-O is shortened by Co doping, which is consistent with XRD results.

### 2.2. Morphology and Compositional Analysis

**Figure 3:**
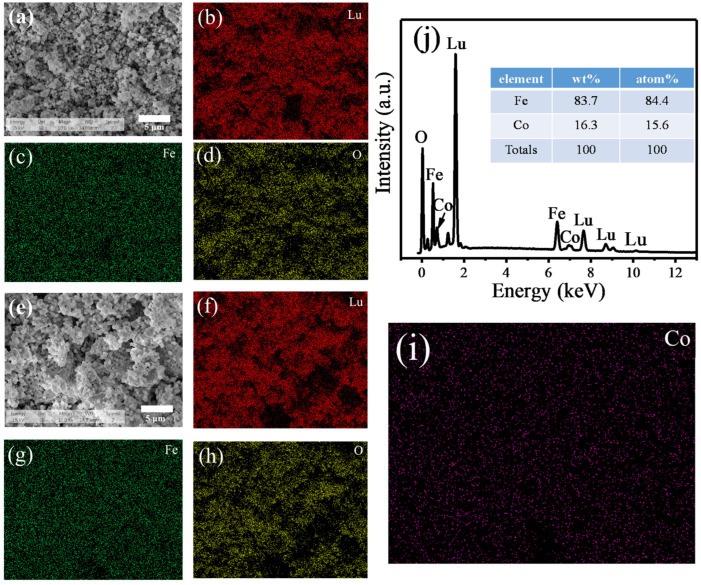
Surface SEM images of LFO (**a**) and LC15 (**e**), and corresponding individual elemental mapping of LFO (**b**–**d**) and LC15 (**f**–**i**); (**j**) EDS spectra of LC15 and the elemental relative content of Fe and Co.

Figure 3 presents the surface SEM images, corresponding individual elemental mapping, and EDS spectra results. Figure 2a,e shows the morphologies of LFO and LC15, respectively. It can be seen that the two samples mainly consist of sphere- or polyhedral-shaped particles, and the average particle sizes of the two samples are 1.8 μm and 2.3 μm, respectively. Obviously, Co doping could lead to the growth of the LuFeO_3_ particles, since a lower reaction temperature and a shorter time of calcination are needed to obtain single-phase Co-doped LuFeO_3_, according to the XRD results, which are in accordance with the results reported by Somvanshi et al. [27]. The individual elemental distributions of LFO and LC15 are displayed in Figure 3b–d and Figure 3f–i, respectively. It shows that all constituent elements are distributed uniformly. The EDS spectrum of sample LC15 shown in Figure 3j manifests that Lu, Fe, Co, and O elements are obviously contained in the sample, and no other impurities were found. As can be seen from the table in the upper right corner of Figure 3j, the contents of the Fe and Co elements are in line with the nominal ratio.

### 2.3. UV–Vis Absorbance Analysis

**Figure 4:**
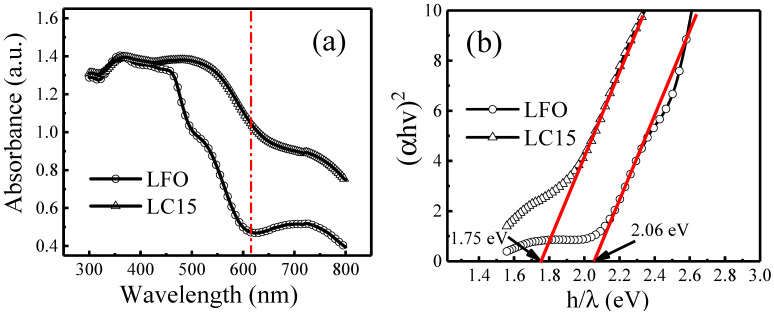
(**a**) UV–vis absorption spectra of samples LFO and LC15, and (**b**) corresponding Tauc plot.

To evaluate the optical absorbance performance of samples LFO and LC15, the UV–vis absorbance spectra were obtained by scanning from 300 to 800 nm using an integrating sphere attachment. On the one hand, as shown in Figure 4a, the Co-doped LC15 sample exhibits a stronger UV–vis absorption than the pure phase LFO sample, which illustrates that visible light utilization efficiency by Co doping can be improved. On the other hand, the wavelength of the absorption edge extends from ca. 600 nm to ca. 700 nm, which means that Co doping can reduce the bandgap (E_g_) and promote the light response range. Based on the above two aspects, the photocatalytic performance should be improved [40].

To quantitatively analyze the effects of Co doping on *E_g_*, the Tauc relationship below was employed to determine the *E_g_* of these two samples.

(1) $$\alpha h\nu =A(h\nu -E_{g}){\;}^{n}\;$$

where *α* is the absorption coefficient, *hν* is the photon energy, *A* is a constant, and *E_g_* is the bandgap [41]. The constant n is determined by the bandgap type of the semiconductor: 0.5 for a direct transition bandgap and 2 for an indirect transition bandgap, where *n* is 0.5 for LuFeO_3_ [42]. The approximated *E_g_* was calculated by the straight-line x-intercept in (*αhν*)^2^ against the *hν* plot transformed from the absorbance spectra, as shown in Figure 4b. The bandgap of the pure phase LFO is 2.06 eV, which is close to the reported value [3,43,44], while the bandgap of LC15 is 1.75 eV. Such a significant decrease in the bandgap was also observed in YbFe_1−x_Co_x_O_3_ (x = 0→0.1) and NdFe_1−x_Co_x_O_3_ (x = 0→0.4) systems [21,28], in which bandgap decreases are reported to be 2.1→1.72 eV and 2.06→1.46 eV, respectively. These results testified that Co doping could effectively tune the bandgap of orthorhombic rare-earth ferrites. One reason for this is that the B cations in ABO_3_ play an important role in the bandgap, and replacing B by more electronegative transition elements could reduce the bandgap [45,46,47]. In this work, the Pauling electronegativity number of Co (1.88) is higher than that of Fe (1.83), which might be responsible for the decrease in the bandgap. Second, the decreased bond length of Fe-O by Co doping enlarges the value of one electron’s bandwidth (W). As reported, the increased W decreases the value of the bandgap [48]. Another reason may be due to the weak orbital hybridization of Co 3d and O 2p states, which leads to less binding energy compared to Fe 3d states [21].

### 2.4. Photocatalytic Degradation of MO under Visible Light Irradiation

**Figure 5:**
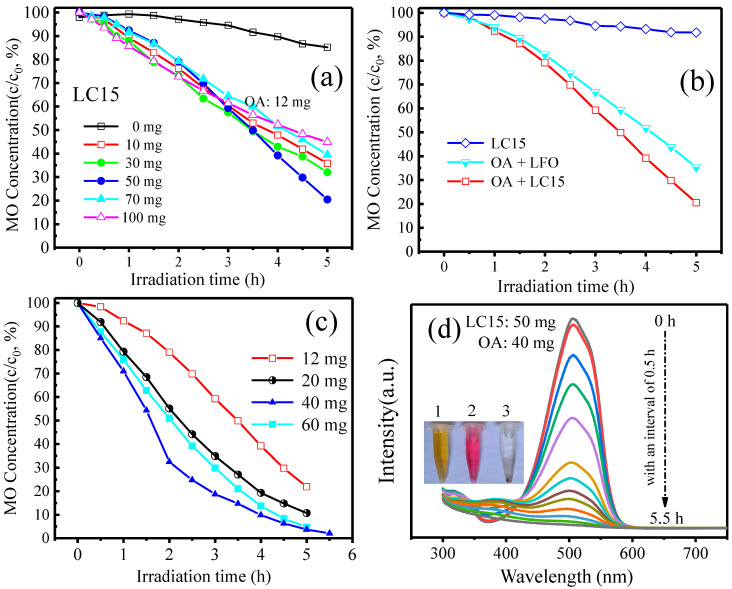
(**a**) Effects of sample LC15 dosage on the photocatalytic degradation of MO with an OA dosage of 12 mg; (**b**) comparison of the photocatalytic capacities of sample LC15 (50 mg) and LFO (50 mg); (**c**) effects of OA dosage on the photocatalytic degradation of MO with an LC15 dosage of 50 mg; (**d**) evolution of the absorbance spectra of MO of the best results with sample LC15 and OA dosages of 50 mg and 40 mg, respectively. The three centrifugal tubes marked by 1, 2, and 3 denote the MO solution, the MO and OA mixed solution, and the degraded solution after 5.5 h of irradiation.

To investigate the photocatalytic capacity of Co-doped LuFeO_3_ particles assisted by OA, experiments were carried out by analyzing the degradation activities of MO solution with an initial concentration of 10 mg/L. OA, a commonly used and environmentally friendly hole scavenger, can be found in most organisms on earth, such as fungi, bacteria, plants, animals, and humans [49]. For an effective photocatalytic process, an optimized catalyst dosage is paramount. Figure 5a shows the change in normalized MO concentration with irradiation time when the dosage of the catalyst LC15 was changed from 0 to 100 mg under the conditions of an initial MO concentration of 10 mg/L and an OA dosage of 12 mg. It can be seen that the MO concentration without a catalyst only decreased by 15% after 5 h of irradiation, while the MO concentration decreased sharply by 64% after adding 10 mg of catalyst. However, as the dosage of catalyst continued to increase, the reduction rates of MO concentration gradually decreased in the cases of 30 and 50 mg of catalyst added. When the dosage of catalyst reached 50 mg, the MO concentration decreased by up to 80%. As the dosage of catalyst continuously increased to 100 mg, the reduction in MO concentration did not increase but rather decreased to 55%. Therefore, 50 mg was considered the optimized catalyst dosage for further photocatalytic experiments. The above phenomenon is reasonable. On the one hand, the intermediate products generated during the photocatalytic reaction process may compete with MO for the active sites on the surface of the catalyst [40], and this competition would become more intense with the increase in the dosage of catalyst. On the other hand, excess catalyst will increase the opacity of the solution, resulting in high light reflectivity, which reduces the absorption of light [50].

Figure 5b presents the comparison of the photocatalytic capacities of LFO and LC15 samples. It is observed that the MO concentration only decreased by 8% after 5 h of irradiation for sample LC15 without OA, due to the low photonic efficiency of LuFeO_3_ resulting from a rapid recombination of photogenerated electron–hole pairs [51]. However, with the assistance of OA, the MO concentration decreased by 65% for the sample LFO. Such a large increase in the photodegradation rate indicates that OA has a great impact on the photodegradation capacity of LuFeO_3_. In addition, compared with sample LFO, sample LC15 can further reduce the MO concentration by 80%, showing an even stronger photocatalytic capacity. It is well known that the particle size of catalysts has an important effect on photocatalytic performance. Generally speaking, the smaller the particle size, the higher the number of active sites of the catalyst that can participate in photocatalysis and the stronger the catalytic capacity [52]. In our case, the LFO sample has a smaller particle size than the LC15 sample, whereas the photocatalytic performance is weaker than that of the LC15 sample. However, according to the UV–visible absorption results, Co doping can improve the utilization of visible light by increasing the absorption and broadening the spectral response range. Thus, combined with the SEM and UV–visible absorbance observations, it is concluded that the influence of optical properties on the catalytic results is more important than that of particle size in our case [40].

To further optimize the degradation conditions, photocatalytic experiments were conducted by varying the OA dosage from 12 to 60 mg, with the LC15 dosage at 50 mg and the initial MO concentration at 10 mg/L. The results are presented in Figure 5c. It reveals that an excess dosage of OA may lead to the occupation of active sites at the surface of sample LC15, leaving no available sites for photocatalysis [53], and 40 mg of OA is the optimized dosage in our system. The MO concentration can be almost completely degraded (~98%) after 5.5 h of irradiation under the conditions of an LC15 and OA dosage of 50 mg and 40 mg, respectively. Figure 5d displays the evolution of the UV–visible absorption spectra of MO and OA mixed solutions with an interval of 0.5 h after 5.5 h of irradiation, which testifies to the good photocatalytic performance of Co-doped LuFeO_3_ particles assisted by OA.

### 2.5. Possible Degradation Mechanism

**Figure 6:**
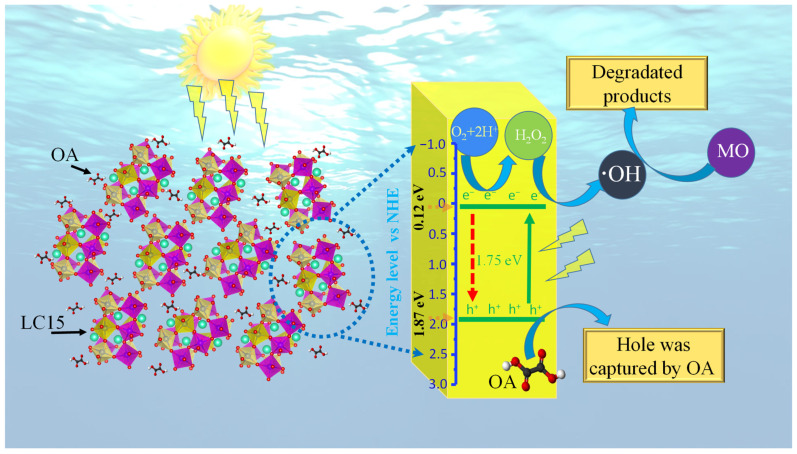
Schematic of the possible mechanism for the photoexcited electron–hole separation and transport processes at the LC15 sample interface, assisted by OA under visible light irradiation.

Based on the above results, a possible degradation mechanism is proposed to explain the enhanced photocatalytic capacity of the Co-doped LuFeO_3_ sample LC15, as illustrated in Figure 6. Since the redox potentials play a key role in the photocatalytic reaction, it is necessary to determine the effect of Co doping on the conduction band minimum (CBM) and valence band maximum (VBM) potentials of LuFeO_3_. These energy levels can be calculated by empirical equations [12,54], as follows:

(2) $$E_{CB}=\chi -E^{e}-0.5E_{g}$$

(3) $$E_{VB}=E_{CB}-0.5E_{g}$$

where *E_CB_* and *E_VB_* are the CBM and VBM potentials, *E^e^* is the energy of free electrons versus hydrogen (4.5 eV), and *E_g_* is the bandgap energy. Finally, *χ* is the electronegativity of a semiconductor, defined as the geometric mean of the electronegativity of the atoms constituting the semiconductor [55], while the electronegativity of an atom is the arithmetic mean [56] of its electron affinity [57,58] and first ionization. The *χ* value of sample LC15 was estimated to be 5.50, slightly larger than that of sample LFO (5.49) [3]. Thus, the CBM and VBM potentials of LC15 are calculated to be 0.12 and 1.87 eV versus the normal hydrogen electrode (NHE), respectively, as indicated by the orange dotted arrows on the blue scale axis in Figure 6. More recently, Zhou et al. [3] found that ·OH radicals are the dominant active species responsible for the dye’s photocatalytic degradation by LuFeO_3_ particles. Further, in the process of photocatalytic degradation of dye in aqueous solution by a semiconductor, it was found that three redox paths that could produce the free radical ·OH are H_2_O/·OH, OH^−^/·OH, and O_2_/H_2_O_2_, and their redox potentials are +2.72, +1.89, and +0.695 eV, respectively [59,60,61]. However, according to the thermodynamic theory, only the O_2_/H_2_O_2_ redox path can occur considering the CBM and VBM potentials of sample LC15, as shown in Figure 6. The related reduction paths are listed below:

(4) $$LC15+hv\;\to \;LC15\left(e_{CB}^{-}\right)+LC15\left(h_{VB}^{+}\right)$$

(5) $$LC15\left(2e_{CB}^{-}\right)+O_{2}+2H^{+}\;\to \;LC15+H_{2}O_{2}$$

(6) $$LC15\left(e_{CB}^{-}\right)+H_{2}O_{2}\to \;LC15+\cdot OH+OH^{-}$$

The oxidation paths of OH^−^/·OH are absent in those of sample LC15 compared with the photocatalytic process of LFO [3]. Further, according to the SEM results, the specific surface area of sample LC15 is smaller than that of LFO. However, the degradation efficiency of sample LC15 is still ca. 23% higher than that of LFO. All these results strongly indicate that the enhancement of the light utilization efficiency by Co doping plays a dominant role in improving the photocatalysis.

**Figure 7:**
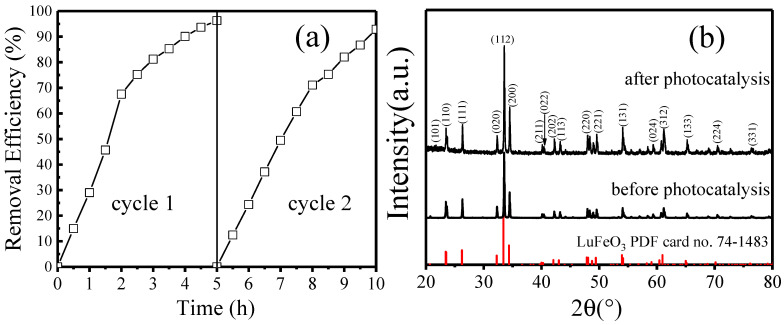
(**a**) Comparison of the removal efficiency between cycles 1 and 2; (**b**) XRD patterns of LC15 before and after photocatalysis.

To reduce the cost of photocatalysis, reusability and stability are two essential features of a photocatalyst. Using the same protocol, the degradation of MO was carried out again by reusing the sample LC15 collected after the first photocatalysis experiment. The comparison of removal efficiency between cycles 1 and 2 is shown in Figure 7a. It is clear that the reduction in removal efficiency is negligible, which suggests that the reusability is good. Figure 7b shows the XRD patterns before and after photocatalysis. Obviously, the orthorhombic structure of sample LC15 is maintained, as all the diffraction peaks corresponding to each plane of orthorhombic LuFeO_3_ are unchanged and no other diffraction peaks appear. These results indicate the good reusability and stability of sample LC15.

## 3. Experimental Section

### 3.1. Chemicals

Highly purified lutetium oxide (Lu_2_O_3_, 99.99%), ferric sesquioxide (Fe_2_O_3_, 99.9%), and cobalt oxide (CoO, 99.5%) were purchased from Aladdin Chemistry Co., Ltd. (Shanghai, China). MO (C_14_H_14_N_3_N_a_O_3_S) was purchased from Shanghai Titan Scientific Co., Ltd. (Shanghai, China). OA (C_2_H_2_O_4_·2H_2_O) was purchased from Tianjin Damao Chemical Reagent Factory (Tianjin, China). All the chemicals are of analytical grade or better and were used as received without further purification.

### 3.2. Preparation of B-Site Co-Doped LuFeO_3_ Powders

B-site Co-doped LuFeO_3_ powders were prepared by the MAS method reported in our earlier work [33]. Specifically, 30 mmol of desired stoichiometry of Lu_2_O_3_, Fe_2_O_3_, and CoO were slowly added to a 50 mL tungsten carbide–cobalt (WC–Co) pot containing WC-Co milling balls with 30 mL of alcohol. Then, the mixture was blended by a planetary mill machine at a rotational frequency of 586 min^−1^ for 12 h. Next, the mixture with the pot open was transferred to a drying oven and heated at 50 °C for 3 h, and then at 150 °C for 24 h. The dried mixture was sequentially mechanochemically activated at a rotational frequency of 456 min^−1^ for 5 h. Afterward, the powder was calcined at 1000–1300 °C for 2–10 h in a muffle furnace. The final Co-doped LuFeO_3_ powders were obtained by grounding the calcinated powders thoroughly in an agate crucible.

### 3.3. Degradation of MO under Visible Light Irradiation

The photocatalytic activities of LuFeO_3_ (LFO) and LuFe_0.85_Co_0.15_O_3_ (LC15) powders were evaluated by the degradation of MO under visible light irradiation. First, the desired amount of OA was dissolved completely in 100 mL of MO solution (10 mg/L). Second, the mixed solution was transferred to a 250 mL jacketed double-layer glass beaker reactor with the required amount of photocatalysts. After reaching an adsorption–desorption equilibrium by stirring for 30 min in the dark, the photocatalytic reaction under stirring was initiated under the irradiation of a 300 W xenon lamp (MC-PF300, Beijing Merry Change Technology Co., Ltd., Beijing, China) equipped with a 420 nm UV-cut filter positioned 8 cm above the reaction solution. The whole process was operated at 20 °C by circulating thermostatic water and stirring at 450 rpm. During irradiation, a small amount of solution (2 mL) was sampled every 30 min. After the solution was centrifugated at 5000 r/min for 3 min, the supernatant was taken, and its absorbance at 505 nm was measured by a UV–vis spectrophotometer (Agilent Carry 60, Santa Clara, CA, USA) to determine the relative concentration of MO. The normalized MO concentration is defined as *I_t_*/*I*_0_×100%, where *I*_0_ and *I_t_* are the absorbance at 505 nm at the beginning and after a certain time of irradiation.

### 3.4. Characterization

The phase purity and structure of the as-prepared samples were investigated by a powder XRD instrument (Bruker D8 Advance, Saarbrucken, Germany) using a Ni-filtered Cu Kα (λ = 1.5406 Å) radiation source at 40 kV and 40 mA with a step size of 0.01°. The IR spectroscopy transmittance measurements were performed over a frequency range of 400 to 4000 cm^−1^ using a FTIR spectrometer (Thermal Nicolet Summit, Waltham, MA, USA) with the KBr pellet technique. Morphological and EDS analyses were measured by field-emission SEM (FESEM) (TESCAN Mira4, Brno, Czech). The absorbance measurements were conducted using a UV–visible spectrophotometer (Shimazu SolidSpec-3700, Kyoto, Japan) with an integrating sphere attachment.

## 4. Conclusions

B-site Co-doped LuFeO_3_ powders were successfully prepared by a MAS method. The required calcination temperature was lower, and the holding time was shorter than that of the parent phase, LuFeO_3_. A shrinkage in lattice parameters was observed in Co-doped LuFeO_3_ samples by fitting the XRD patterns, indicating a successful substitution of Co at Fe sites. FTIR results revealed that two bands attributed to the Fe-O stretching and bending vibrations of the FeO_6_ octahedral groups both blueshifted in accordance with the XRD results. The particle size of LC15 is slightly larger than that of LFO. The UV–vis absorbance spectra show that Co doping of LuFeO_3_ can considerably improve the light utilization efficiency by increasing the absorption and broadening the spectral response range. The sample LC15 shows a 23% higher photocatalytic capacity than that of the sample LFO and can decrease the MO concentration by 98% under optimized conditions for 5.5 h of visible light irradiation. A possible degradation mechanism is proposed. In summary, our results clarify that Co doping can improve the photocatalytic performance of orthorhombic LuFeO_3_.

## Figures and Tables

**Table 1:** Refined structural parameters for LuFe_0.85_Co_0.15_O_3_ and LuFeO_3_ samples (error in 10^−4^ order in refined parameters).

Sample Space Group Rwp χ^2^	Lattice Parameters (Å)	Atoms	Positions				Volume (Å^3^)	Angle Fe-O-Fe
			Wyckoff	x	y	z		
LuFe_0.85_Co_0.15_O_3_ Pbnm 8.94 1.3	a = 5.1976 ± 1	Lu	4c	0.9800	0.0714	0.25	216.5313 ± 24	
	b = 5.5373 ± 1	Fe(Co)	4b	0	0.5	0		
	c = 7.5235 ± 1	O1	4c	0.1190	0.4539	0.25		140.601
		O2	8d	0.6893	0.3071	0.0621		142.406
LuFeO_3_ [33] Pbnm 7.74 1.46	a = 5.2154 ± 1	Lu	4c	0.9792	0.0715	0.25	219.0780 ± 35	
	b = 5.5537 ± 1	Fe	4b	0	0.5	0		
	c = 7.5637 ± 1	O1	4c	0.1179	0.4579	0.25		141.632
		O2	8d	0.6890	0.3050	0.0638		142.121

## Data Availability

Data supporting the results can be provided by the corresponding author via email.
